# Expanding the geographical distribution of *Blastobotrysmalaysiensis* (Saccharomycetales) beyond the Asian continent – a cave fungus first reported in the Americas

**DOI:** 10.3897/BDJ.10.e80226

**Published:** 2022-11-15

**Authors:** Antônio Sérgio Ferreira de Sá, Lucas Leonardo-Silva, Solange Xavier-Santos

**Affiliations:** 1 Universidade Estadual de Goiás, Campus Central, Laboratório de Micologia Básica, Aplicada e Divulgação Científica (FungiLab), Anápolis, Brazil Universidade Estadual de Goiás, Campus Central, Laboratório de Micologia Básica, Aplicada e Divulgação Científica (FungiLab) Anápolis Brazil

**Keywords:** Saccharomycetales, geographic distribution, yeast, cave, phylogeny

## Abstract

**Background:**

Saccharomycetales are ascomycetic yeasts and, amongst them, the genus *Blastobotrys* has approximately 30 known species. *Blastobotrysmalaysiensis* is a yeast species, described from cave samples, known until then only from Malaysia. In this study, we characterise a new strain and report the second occurrence record of this species. Here, *Blastobotrysmalaysiensis* URM 8507/SXS 675, was collected from sediments samples from a cave in the Parque Estadual de Terra Ronca (PETER) in Goiás, Brazil. Phylogenetic analyses revealed strong support with the sequence of the species type, as well as with other species of the clade. This new record contributes by providing new molecular data for the species and expanding the knowledge of its distribution beyond the Asian continent.

**New information:**

First record of a yeast for the American continent and its second mention for the world.

## Introduction

The order Saccharomycetales comprises the ascomycete yeasts, with about 1000 described species. They can be found in various niches, either as saprotrophs, in mutualistic associations with plants and animals and even as pathogens (Suh et al. 2007).

*Blastobotrys Klopotek (1967)* is a genus of this order and has approximately 30 known species. The genus is characterized by the presence of setae, such as cell wall projections, micropores in the septa, central micropores and the formation of blastoconidia that form in denticles. Dimorphism is also observed in several species of the genus and can be found either as a filamentous structure (mycelium) or in yeast-like growth (unicellular), with different dimorphic mechanisms for each species (Malak et al. 2016).

This genus forms a clade closely related to three other yeast genera (*Candida*, *Arxula* and *Sympodiomyces*), also presenting the genus *Trichomonascus* as an ascosporic state (Kurtzman and Robnett 2007). Also, phylogenetic data showed that *Blastobotrys*, *Sympodiomyces*, *Arxula* and some *Candida* species correspond to a single genus, defined as *Blastobotrys* and that species of *Arxula* and *Sympodiomyces* should be transferred to *Blastobotrys* (Kurtzman and Robnett 2007).

*Blastobotrys* comprises species with diverse niches, with strains isolated in different habitats, such as soil and plants (Thomas et al. 2019) and even urban (Vanderwolf 2021) and wild animals (Bhadra 2008). Even Blastobotrys species have been described from cave samples, such as B. chiropterorum, isolated from the liver of a cave bat in Colombia (Grose and Marinkelle 1968). With promising species for biotechnological applications, such as *B.adeninivorans* and *B.raffinosifermentans*, which are thermotolerant and xerotolerant because they produce and store lipids at high temperatures (Thomas et al. 2019). *Blastobotrysmalaysiensis* was described by Kurtzman (2007) in a cave in Malaysia at the yeast stage. Since its description, there have been no further reports of the occurrence of this species, resulting in little information on its ecology and distribution available in the literature. This study is part of a broad survey of the mycobiota of karst caves in Central Brazil and aims to characterize a new strain and report the second occurrence record of this species, expanding the knowledge of its distribution beyond the Asian continent.

## Materials and methods

### Study area

**Figure 1.**
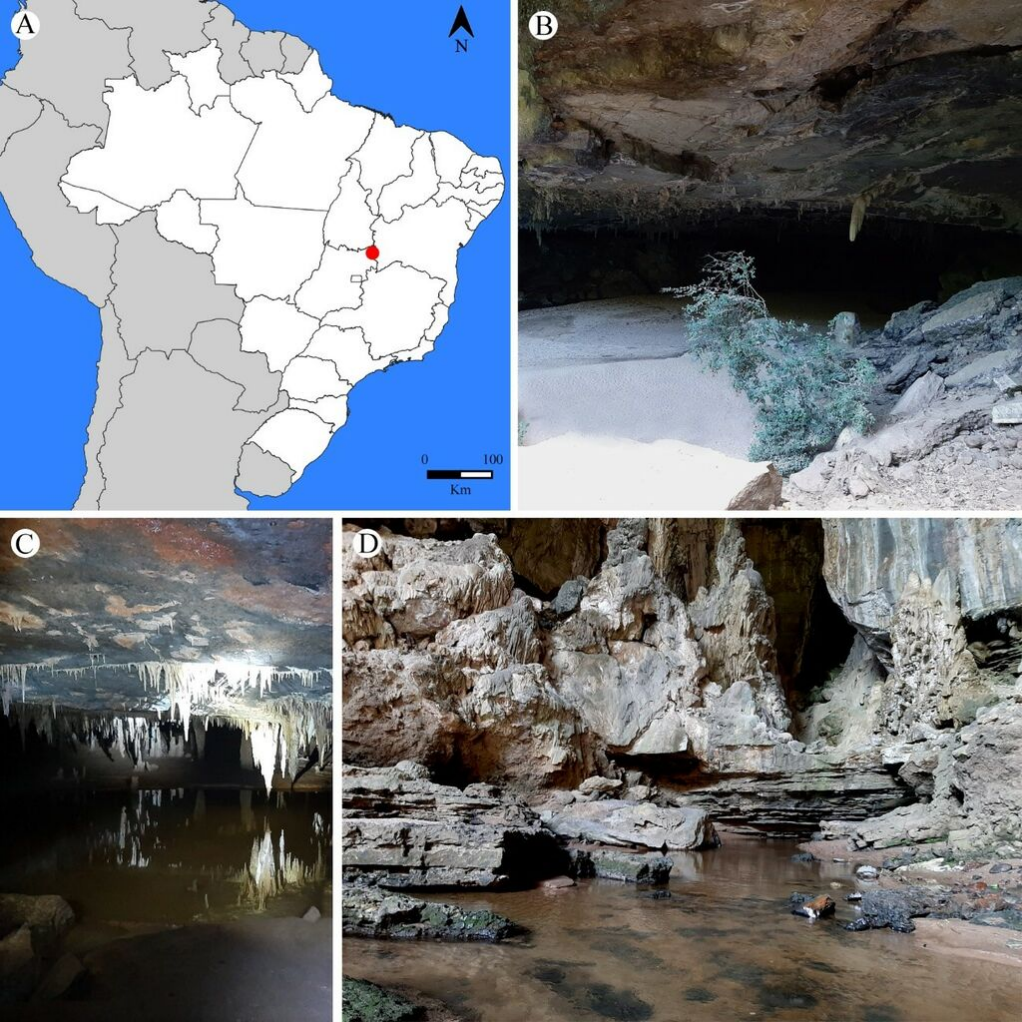
**A** Location of the studied area, Parque Estadual de Terra Ronca (PETER), Goiás, Brazil; **B** Entrance of Lapa do Angélica cav;e **C** Internal part of cave (aphotic zone); **D** Resurgence.

The material studied was isolated from sediment samples of the Angélica cave (-13.5173, -46.388077), located in the Terra Ronca State Park (PETER), in the municipality of São Domingos, far east of the state of Goiás, bordering the state of Bahia, Brazil (Fig. 1). This cave has an extension of 14,100 m, being amongst the largest in the country (Matteucci 2001), formed in carbonate rocks, this cavity is traversed in all its extension by the river Angélica.

The PETER covers three Brazilian regions and its predominant biome is the Cerrado. The area comprises 57,000 hectares, with a climate of type AW (Tropical Savanna), with cold and dry rains in winter and hot humid summers and average annual precipitation of 1,500 mm (Koppen 1948). PETER has an important speleological complex in South America; in it lies part of the region known as the "Bambuí Speleological Province or Bambuí Group", characterized by the outcropping of carbonate rocks, being the karstic region among the 19 found in Brazil, with the largest number of known caves (CECAV (Centro Nacional de Pesquisa e Conservação de Cavernas) 2019).

### Sampling and isolation

The specie reported here was isolated from sediment samples from Lapa do Angélica Cave, specifically from the aphotic zone, with only one strain. We observed that, in these sediments, there were signs of bat guano, in small quantities. The isolation was performed by contacting the swab soaked in sterile saline solution (0.9%) with the sediment and then streaked on Petri plates containing medium Sabouraud (Sa) Agar, increased with chloramphenicol (15 mg l^1^). The plates were sealed with film paper and transported to the laboratory of Basic, Applied and Scientific Dissemination Mycology (FungiLab) of the State University of Goiás, Central campus, where they were incubated at 28℃, the temperature verified in the cave during isolation, in aerobiosis for seven days. Grown colonies were isolated and purified on Potato-Dextrose-Agar (PDA) medium.

After obtaining the pure colony, a 5 × 5 mm inoculum was removed and inoculated into an erlenmeyer flask containing Yeast-Peptone-Dextrose (YPD) broth and incubated under constant agitation (130 RPMs) at a room temperature (± 28℃), to be used in the assimilation and fermentation experiments. In addition, inocula from the pure colony were subjected to growth at different temperatures (25, 28, 30, 37 and 40ºC) and in different culture media, such as PDA, Malt Extract Agar (MEA) and Mycosel Agar, the latter being used to verify resistance to cycloheximide. For morphological characterisation, light microscopy (OLYMPUS CX31) was performed using cotton blue lactophenol and sterile water to prepare the slides, where it was possible to observe the microstructures. The purified colonies were stored in mineral oil and deposited in the culture collections of the URM Micoteca (URM 8507) and the ueg FungiLab, under voucher SXS 675 using the Castellani method.

### Assimilation and fermentation test

The assimilation and fermentation tests were performed with five sugars: xylose, glucose, maltose, lactose and galactose. The isolate of *B.malaysiensis* was inoculated in 5 ml of basal medium (Peptone and Yeast extract) increased with 2% of each sugar (carbon source) and incubated at 27 and 30℃ for 8 days.

The assimilation of the carbon sources was considered positive when the presence of cell mass was observed, verified according to the concentration of cells, through the optical density spectrophotometric method (OD 600). For the fermentation test, the Durham tube technique was used, being considered positive fermentation when half of the tube was filled with gas.

### DNA extraction, PCR amplification and Sequencing

For taxonomic identification, a 0.5 ml of cell mass was collected from the culture in YPD broth and submitted to DNA extraction using the CTAB method (Goés-Neto 2005, Hosaka 2009). After genomic DNA was obtained, the ITS (Internal Transcribed Spacer) ribosomal nuclear region was amplified from primers ITS5 / ITS4 (White et al. 1990), using DNA Engine Tetrad 2 Peltier Thermal Cycler (BIO-RAD), with initial denaturation at 90ºC for 5 minutes and then 35 cycles of denaturation at 95ºC for 30 seconds, the annealing occurred at 55ºC for 30 seconds, extension at 72ºC for 1 minute; the reaction ended with a final extension of 7 minutes at 72°C and storage to 4°C. The amplification product was purified using the Multiscreen filter plate (Millipore Corp.). Sequencing was performed from the same primers used in amplification, performed by Macrogen Inc. (Seoul, South Korea).

### Phylogenetic analysis

The sequences obtained, as well as the sequences retrieved from GenBank (NCBI), shown in Table 1, were combined and aligned in MAFFT 7 (Katoh et al. 2017). The alignments were analyzed and minor adjustments were performed manually with MEGA 6 (Tamura et al. 2011). The sequences used in this analysis correspond to species and genera closely related to *Blastobotrys*, according to (Kurtzman 2007). The new sequence obtained here has been deposited in GenBank under accession number MZ702867. *Schizosaccharomycesjaponicus* was used as an outgroup for phylogenetic inferences.

We used two different analyses, Maximum Likelihood (ML) and Bayesian Inference (BI). ML was performed from TOPALi v.2 (Milne and Lindner 2009) determined by 1,000 bootstrap replications, resulting in the branching support value (BS), whereas BI was conducted using MrBayes 3. 2.7 (Ronquist 2012), with runs performed with 2,000,000 generations, the convergence and stability of the runs were evaluated from the average standard deviation (> 0.01) in Tracer v.1 .6, as well as the calculation of the Baysesian posterior probability (BPP).

### Materials

Enter subsection text

## Taxon treatments

### Blastobotrys malaysiensis

Kurtzman, 2007

B0E84A0E-5CE8-5458-BDD3-35D52E4D7991

#### Materials

- **Type status:** Other material. **Occurrence:** occurrenceDetails: Isolated from cave sediments; recordNumber: URM 8507/SXS 675; associatedSequences: MZ702867; occurrenceID: 0CC4B08A-70E3-5DE6-9209-1F6A530E0BBF; **Taxon:** order: Saccharomycetales; scientificNameAuthorship: Blastobotrysmalaysiensis Kurtzman, 2007; **Location:** higherGeography: South America: Brazil: Goiás: Parque Estadual de Terra Ronca; continent: South America; country: Brazil; countryCode: Brazil/BR; stateProvince: Goiás; municipality: São Domingos de Goiás; locality: Cave Lapa do Angélica; decimalLatitude: -13.5173; decimalLongitude: 46.388077; **Identification:** identifiedBy: Sá-Ferreira A.S., Leonardo-Silva, L. Xavier-Santos, S.

#### Description

At five days of growth at 25, 28 and 30℃, in PDA medium, the colony showed opaque white colouration, with a mycelial fringe and lobed margin; when growing in MEA, at 27ºC, yellowish colony, with cottony aerial mycelium in the centre and dense and opaque margin was observed. In both media, growth of septate hyphae and pseudohyphae was noted. In MEA medium, in samples from the margin of the colony, abundant spherical cells (2.74 - ˗4.50 µm) with multilateral budding were observed (Fig. 3A˗ B); blastoconidia were also observed, formed from small pedicels (Fig. 3C˗ D). In our cultures, ascospore production was not observed. At 37ºC, this strain showed good growth on MEA and Mycosel agar, with a yellowish-white colony of dense aspect after seven days more abundant yeast cells were observed, with few pseudohyphae and setae. At 40ºC, in the same culture media, after 10 days of incubation, the colony grew less than 0.5 cm beyond the inoculum, presenting a yellowish color, with a wrinkled aspect.

##### Habitat and distribution

Isolated from cave sediments environments. The current knowledge about its distribution reveals that the species is restricted to tropical environments, with only two records: Malaysia (Kurtzman 2007) and Brazil (this study).

##### Note

*B.Malaysiensis* showed extensive growth at 37ºC, with discrete development at 40ºC. Besides to growing on medium supplemented with cyloheximide. We observed that temperature did not affect the fermentative capacity of *B.malaysiensis*, as the results were the same regardless of the temperature (27 or 30ºC) (Table 2).

## Analysis

### Molecular phylogeny

**Figure 2.**
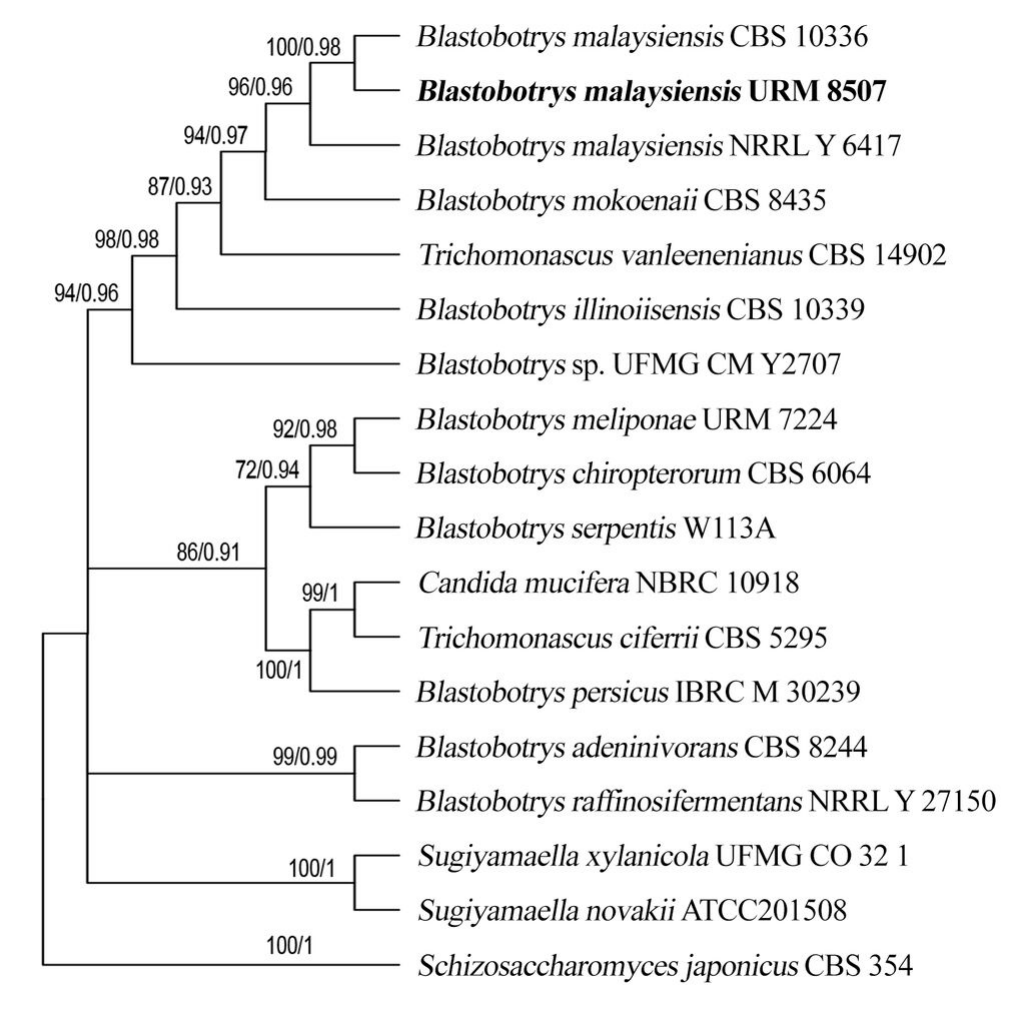
Phylogenetic relationships between *Blastobotrysmalaysiensis* URM 8507/SXS 675 (in bold) and other *Blastobotrys* species and corresponding clade, based on rDNA of the ITS (Internal Transcribed Spacer) region. Values at nodes indicate bootstrap from Maximum Likelihood/Bayesian posterior probability analysis. *Schizoaccharomycesjaponicus* was included as an outgroup.

**Figure 3.**
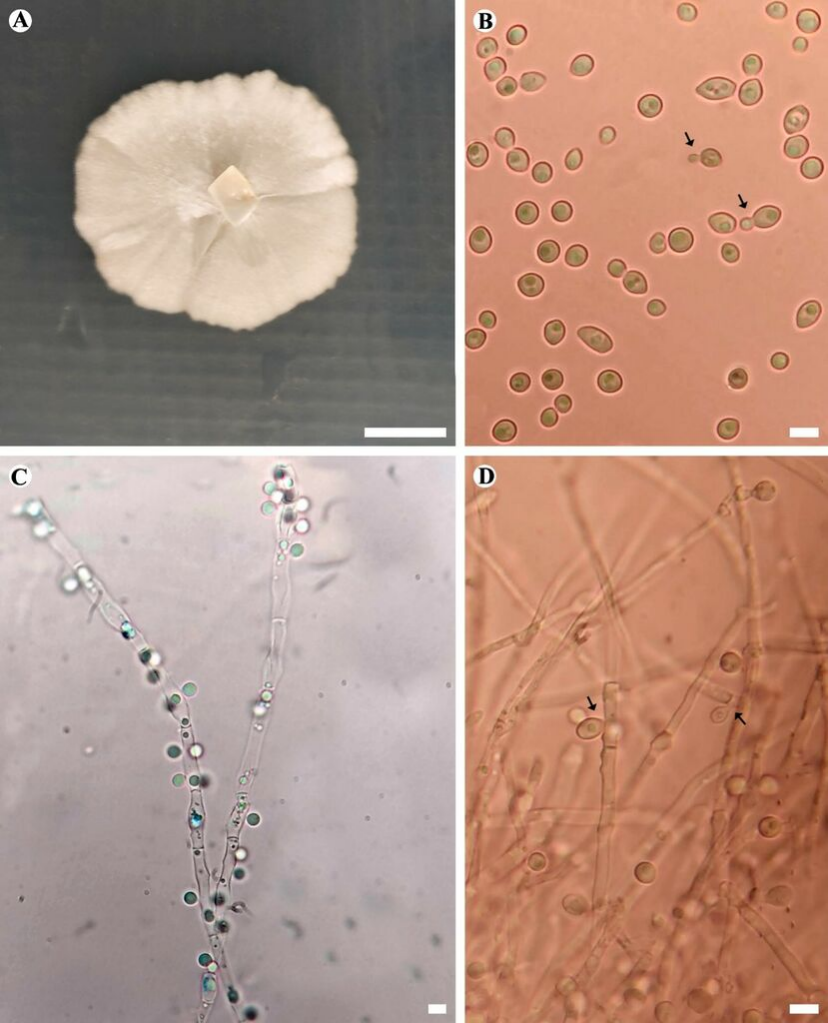
Morphology of *Blastobotrysmalaysiensis* (URM 8507/SXS 675). **A** Colony of *B.malaysiensis*; **B** Yeast cells, with multilateral budding (arrows); **C** Blastoconidia stained with lactophenol blue; **D** Blastoconidia attached to hyphae from pedicels (arrows). A-B grown on PDA (Potato Dextrose Agar) after eight days of growth at 27ºC and C-D grown on MEA (Malt Extract Agar) at 27ºC for five days. Scale bars: 10 mm (A), 10 µm (B, C, D).

The dataset included sequences from 20 yeast species that are related to the *B.malaysiensis* clade, according to Kurtzman (2007). The two analyses resulted in similar topology, however, only the Bayesian topology is shown (Fig. 2) and the statistical values (BS/ BPP), respectively, are indicated for each node. The evolutionary model, used in the ML and IB analyses, was TVM+G, based on the AIC (Akaike Information Criterion) criteria. *Blastobotrysmalaysiensis* (URM 8507/SXS675) showed strong support (BS = 100%, BPP = 0.98) clustering close to the type species (CBS10336), with 100% similarity.

## Discussion

As verified by Kurtzman (2007) in the Asian strain, we found that the South American strain of *B.malaysiensis* (URM 8507/SXS 675) also showed resistance to cycloheximide, as well as growth at 37°C and 40℃. This thermotolerant characteristic is well understood and observed in several species of the genus, which makes it considered biotechnologically promising (Sanya et al. 2021). The fermentative characteristics also coincide with those found in the description of this species, and, in addition to data from Kurtzman (2007), we tested the ability of *B.malaysiensis* to ferment glucose. We observed that the strain fermented little of this sugar under the conditions presented, corresponding to less than half of the gas occupying the Durham tube.

The strain reported here was isolated in the cave's resurgence, an area that is not open to tourists, as it is difficult to access. This access is made either externally, through a 10 km trail in a dense forest or internally, through the river inside the cave, a route that presents great obstacles, considered very dangerous by regional guides and the speleological community. For this reason, it is an environment that has suffered little impact from human visitation.

Some hypotheses may explain how this yeast was dispersed to this specific environment since, until now, it was only reported occurring in a cave environment in Malaysia. Zhang et al. (2018) state that fungal species diverged long before the formation of karst caves, which refutes the hypothesis that these species are troglobic.

Thus, we cannot assume that *B.malaysiensis* is a troglobic yeast, despite only being known in cave environments, but we emphasize the importance of further research efforts involving this species, to elucidate its current distribution. Whether it is a species restricted to subterranean environments or if this current distribution is only due to the lack of sampling and precise taxonomic identification. The present study reports the second worldwide occurrence of *B.malaysiensis*, expanding its distribution beyond the Asian continent.

## Supplementary Material

XML Treatment for Blastobotrys malaysiensis

## Figures and Tables

**Table 1.** List of species, strains and GenBank accession code for ITS sequences used in phylogenetic analyses.

**Species**	**Strain/Specimen No.**	**Country**	**GenBank accesion N^o^ (ITS)**	**Reference**
*Blastobotrysmalasyensis*	CBS: 10336	Malaysia	NR_165958	Vu 2016
*Blastobotrysillinoisensis*	CBS: 10339	EUA	NR_165957	Vu 2016
*Blastobotrysadeninivorans*	CBS: 8244	Netherlands	EU343811	GenBank
*Blastobotryschiropterorum*	CBS: 6064	Colombia	KY101750	Vu 2016
*Blastobotrysmalasyensis*	NRRL Y-6417	-	DQ898170	Kurtzman 2007
*Blastobotrysmalaysiensis*	URM 8507	Brazil	MZ702867	This study
*Blastobotrysmeliponae*	URM 7224	Brazil	KT448719	Crous 2016
*Blastobotrysmokoenaii*	CBS: 8435	South Africa	KY101754	Vu 2016
*Blastobotryspersicus*	IBRC-M 30239	Iran	KY352042	Nouri et al. 2017
*Blastobotrysraffinosifermentans*	NRRL Y-27150	-		Kurtzman 2007
*Blastobotrysserpentis*	W113A	India	AM410670	Bhadra 2008
*Blastobotrys* sp. E4	UFMG-CM-Y2707	Brazil	KT377031	GenBank
*Candidamucifera*	NBRC 10918	Brazil	LC158135	Tsang 2017
*Schizosaccharomycesjaponicus*	CBS: 354	Japan	AB243296	GenBank
*Sugiyamaellanovakii*	ATCC201508	-	LC120357	Tanahashi and Hawes 2016
*Sugiyamaellaxylanicola*	UFMG-CO-32.1	Brazil	KC493642	Morais et al. 2013
*Trichomonascusciferrii*	CBS: 5295	-	NR_111160	Schoch et al. 2014
*Trichomonascusvanleenenianus*	CBS: 14902	Netherlands	NR_168170	Groenewald 2018

**Table 2.** Fermentative and assimilative characteristics of *Blastobotrysmalaysiensis* for five carbon sources. (+) Positive, (*) Poor result.

**Substrate**	**Assimilation**	**Fermentation**
Glucose	+	*
Lactose	+	+
Maltose	+	+
Galactose	+	+
Xylose	+	*
